# The impact of material hardship severity and frequency on health outcomes: Evidence from New York City

**DOI:** 10.1371/journal.pone.0335790

**Published:** 2025-10-30

**Authors:** Trevor Memmott

**Affiliations:** McCormack School, University of Massachusetts Boston, Boston, Massachusetts, United States of America; Sheffield Hallam University, UNITED KINGDOM OF GREAT BRITAIN AND NORTHERN IRELAND

## Abstract

Material hardship – the struggle to meet basic household needs – regularly impacts millions of Americans. Despite a growing body of research on the impacts of economic deprivation, a comprehensive understanding of the ways in which the experience of material hardship impacts health is still lacking in extant literature. To address these shortcomings, this manuscript analyzes data from the NYC Poverty Tracker, a longitudinal survey designed to capture the experience of material hardship among New York City residents, to explore the relationship between five major forms of hardship (food, energy, housing, financial, and medical) and physical and mental health outcomes. Findings show a clear association between all five forms of hardship and diminished health outcomes. Further, the frequency (how often), severity, and experience of facing multiple hardship simultaneously is associated with larger negative impacts on how a respondent rates their physical and mental health. These results offer a more detailed, comparative understanding of the way material hardships intersect with health, suggesting the need for targeted policy responses that not only address immediate deprivation but also consider the broader health implications that results for those living under chronic hardship. The findings offer key insights into the literature on health and social welfare in addition to important evidence for policymakers attempting to alleviate the burden of material hardship and improve public health outcomes.

## Introduction

Recent decades have seen the emergence and growth of the study of material hardship, defined as a household’s struggle or inability to meet its basic needs [1], including inadequate provision of food, housing, healthcare, and energy services. Material hardship scholarship measures and examines these material inadequacies directly, rather than relying on traditional measures of household income or poverty rates, which are often not fully reflective of lived experience.

Material hardship matters not only as an indicator of economic disadvantage, but also as a determinant of health. A growing body of research links hardship to higher rates of adverse mental and physical health consequences, especially among vulnerable populations. Studies of families and their children [2–4], young and working-age adults [5,6], and the elderly [7] have found a consistent association between facing one or more material hardships and health outcomes, such as depression, stress, trouble sleeping, and suicidal thoughts.

While the mental health effects appear to be relatively consistent across hardship indicators, e.g., all contribute to issues like anxiety and depression, the physical health effects may be more specific. Evidence shows, for example, chronic food insecurity contributes to issues of diabetes and hypertension [8], while energy insecurity exposes households to environmental toxins and indoor pollution [9], and health insecurity can exacerbate the physical health conditions an individual already experiences due to delayed or avoided care [10].

While the prevalence of material hardship is well studied, the consequences to health are less comprehensively understood. Much of the extant literature relies on cross-sectional data, limited sample sizes, and/or individual hardships in isolation. Further, little existing research on the relationship between material hardship and health examines hardships comparatively within models (e.g., the relative effect of food versus housing hardship on mental health) or between models (e.g., the relative effect of food hardship on mental compared to physical health).

This manuscript addresses these shortcomings by leveraging data from The New York City Longitudinal Study of Wellbeing – colloquially and hereafter referred to as the “NYC Poverty Tracker” – a longitudinal study of economic disadvantage among New York City residents. Measuring self-reported health outcomes, the analysis examines which hardships are most detrimental to health, finding that all forms of material hardship – food, energy, housing, health, financial, and medical – are strongly associated with worse self-reported physical and mental health outcomes, and that severity, frequency, and simultaneity of hardship matters. These findings provide important insights for policymakers and scholars who hope to understand and address the detrimental impact that material hardship, which regularly impacts tens of millions of American households, can have on public health.

### The prevalence of hardship

Measures of income and poverty are still dominant in scholarship around economic inequality and in public policy where, for example, most federal assistance programs determine eligibility by an individual’s earnings relative to the Federal Poverty Line (FPL). As noted by Thomas [11], these measures of income and poverty index “*likely* experiences of deprivation,” while material hardship scholarship assesses the reality of deprivation, wherein two comparable households with the same level of income may have radically different experiences due to issues like debt accumulation, government transfers, access to credit, familial assistance, higher costs, and/or available assets.

Material hardship, which is most often measured in the literature using five hardship indicators – food, housing, health, energy, and financial – is pervasive in the United States, impacting millions of households daily. Food insecurity, for example, impacts an estimated 12.8% of U.S. households, equating to over 13 million households [12]. Housing instability is similarly prevalent, with around 15% of American renters spending more than 50% of their income on rent, which Housing and Urban Development (HUD) classifies as “severely rent burdened” [13]. Other hardships are also common across the United States; 43% of working aged Americans are classified as uninsured or underinsured [14], 27% of U.S. households report struggling to pay their energy bill or to keep their home at a comfortable temperature [15], and tens of millions of Americans report financial hardship, with 30% reporting being unable to pay their bills in full and 40% being unprepared for a surprise $400 expense [16].

### Material hardship and health

Evidence has shown that material hardship can lead to negative consequences to health, long-term financial stability, and social outcomes [4,17,18]. Individuals who struggle to meet their basic needs, like living in stable housing conditions or having enough food to eat, are more likely to report poor health and psychological distress. These associations have been documented among children, working age adults, and the elderly [5,7,19].

Among adults, material hardship has been significantly associated with lower self-reported health, problems falling and staying asleep, higher rates of depression, stress, and suicidal thoughts [19]. In their study of depression among low-income adults, Heflin and Iceland [4] found that material hardship played the largest mediating role in reported rates of depression after controlling for numerous other sociodemographic factors. Additional scholarship has found that elderly Americans are especially vulnerable to the negative physical and mental health consequences of material hardship because they are more reliant on the healthcare system [20].

Similar findings have been shown across specific domains of material hardship. Children, the elderly, and pregnant women are particularly susceptible to severe negative physical and mental health consequences of food hardship, including diabetes, hypertension, and higher reports of anxiety and depression [21–23]. Housing hardship, which has risen in recent years with an increase in the average rent-to-income ratio (RTI) [24], is associated with physical health maladies the come from living in poor housing conditions [9,25] and mental health stressors resulting from facing the threat of eviction [26,27]. Further, because housing makes up a large share of the expenses for many lower-income households, housing hardship is correlated with other forms of hardship like cutting back on food or skipping health visits [28,29].

Households facing energy hardship, which is most often a chronic condition [30], regularly engage in risky and dangerous “coping strategies” that can have hazardous consequences to mental and physical health, including higher rates of asthma, developmental delays among children, and increased blood pressure [19,31]. Such coping strategies include leaving the home at unsafe temperatures, burning trash, and foregoing other household necessities like food and medicine to keep the power on [32]. Finally, those facing health hardship often go without insurance or remain underinsured, which increases vulnerability to both acute and chronic illness, creating a negative cycle in which medical debt and under-provision of care leads to worsening health outcomes over time [33,34].

### Social determinants of health

The Social Determinants of Health (SDH) framework analyzes the ways in which the socioeconomic conditions that people are born into and live under can impact their health status [35]. These factors include physical environment, employment conditions, social network, and access to healthcare. Marmot [36] details the ways in which material inequalities – e.g., lack of access to quality healthcare, ingestion of mold, regularly being displaced from one’s home, chronically struggling to pay bills – contributes directly to physical health maladies (heart disease, obesity, diabetes, cancer) as well as diminished mental health (stress, anxiety, suicidal ideation). Marmot notes that these effects compound such that an inner-city neighborhood in Baltimore has a life expectancy that is 20 years lower than nearby wealthy neighborhoods.

There are several mechanisms through which each hardship (food, energy, housing, health, and financial) may directly or indirectly impact an individual’s health. For example, food hardship can lead to malnourishment, obesity, and hypertension, but also the stress of worrying about where one’s next meal is going to come from [37]. Further, experiencing hardship may exacerbate pre-existing adverse health conditions, such as lack of healthcare access leaving mental or physical illnesses untreated [38,39]. Finally, multiple hardships may compound to worsen health. For example, those experiencing financial hardship are often forced to live in low-income communities with poor housing conditions, increasing their likelihood of being exposed to both outdoor and indoor pollution and their resulting negative health consequences [40].

All the above mechanisms are likely exacerbated in cases where the experience of material hardship is a chronic condition. For example, scholars have found that chronic lack of access to affordable healthcare leads to health issues that compile and worsen, in addition to the incurring of medical debt which is associated with higher rates of self-reported rates of depression, worry, and anxiety [41,42]. In their review of the health psychology literature, Johnson and Acabchuk [43] note that the stress associated with chronic poverty leads to hormonal imbalances that contribute to a wide range of health disorders including physical (inflammation, immune function) and mental (depression, anxiety) maladies.

The literature on material hardship and the SDH framework highlight pathways by which hardship may influence physical and mental health. While prior research has linked hardships such as food and housing insecurity to adverse health outcomes, most studies examine these hardships in isolation, use cross-sectional data, or study a small sample of respondents. Relatedly, little work attempts to measure not just the severity of hardship, but how hardships accumulate and how frequently they occur. As a result, we lack an understanding of which types of hardship are most detrimental and how multiple hardships may compound to exacerbate diminished health outcomes. This paper addresses these gaps, analyzing a sizable and longitudinal sample of New York City households to measure which hardships are most impactful to physical and mental health, the added effect of chronic hardship, and the cumulative impact of experiencing multiple hardships simultaneously.

## Materials and methods

### Data

Data for this analysis comes from the NYC Poverty Tracker, a longitudinal survey of New York City households collected in collaboration between the Colombia University Center on Poverty and Social Policy and the Robin Hood Foundation. The survey is designed to capture the experience of poverty, material hardship, and health outcomes over time among a representative sample of New York City households as residents enter and exit from poverty. The Poverty Tracker uses stratified random sampling, employing a Random Digit Dial (RDD) approach on both cell phones and landlines of residents over the age of 18, based on sociodemographic group status (age, race, sex, household size), and oversamples those populations most in risk of poverty. The survey is then weighted to account for selection and non-response bias, and post-stratified to match demographic population statistics from the American Community Survey (ACS). Panel weights are applied to account for attrition, and values are imputed for missing observations by means of a two-step multiple imputation with expectation-maximization bootstraps using information on demographics, health, material hardship, finances, housing, and services use frequency [44]. Cohort 2, the longitudinal 6-year panel analyzed in this study, begins with a sample size of 3,908 respondents. The sample size declines to 3,182, 2,952, 2,722, 2,610, and 2,222 in the subsequent 5 years, respectively.

The survey is structured as a baseline instrument with subsequent follow-up surveys at three-month intervals. This analysis utilizes longitudinal data that tracks respondents at yearly intervals from 2015–2020 (baseline, 12 months, 24 months, 36 months, and 48 months). This approach preserves the original sample and keeps questions about hardship and health outcomes consistent in each yearly survey wave. However, except for an item in the Supporting Information, the first year of the survey is excluded in this analysis due to a change in survey methodology which includes different measures of health outcomes.

The NYC Poverty Tracker has been used extensively in scholarly research, including to investigate the distribution of material hardship among wage earners [45], the hardship-mitigating effect of the Child Tax Credit [46], food insecurity among the elderly [47], and risk factors for homelessness [48]. Its extensive and well-validated survey battery on the experience of material hardship, reasonably large sample size, and longitudinal nature allow for a more comprehensive and comparative analysis of the impacts of hardship on health than currently exists in the extant literature.

### Measures

This analysis includes three primary outcome variables. The first is a binary variable, transformed from a Likert scale of respondent’s rating of their general health, that measures whether a respondent is in poor health (“poor” or “fair”) or in good health (“good,” “very good,” or “excellent”). Second is a measure of respondent life rating, where respondents are asked to rate their life overall, on a scale of 0–10, ranging from “the worst possible life overall” to “the best possible life overall.” The final outcome measure is a variable indicating respondent level of distress. This approach was designed to be consistent with the Kessler 6 Scale, created by Robert C. Kessler to designate manifestations of psychological distress [49]. Respondents are asked how often, on a five point scale ranging from “none of the time” to “all of the time,” they felt: nervous, restless or fidgety, hopeless, worthless, that everything was an effort, or so sad that nothing could cheer them up over the last 30 days. The total value of these responses is then summed, ranging from 0 (“none of the time” to each question) to 24 (“all of the time” to each).

The Kessler Scale has been validated extensively for both internal consistency and accuracy in medical illness screening [50–52]. While the established cut-off score denoting serious mental distress is a 13 on the Kessler Scale [49], Prochaska et al. [53] show that a score of 5 and above is correlated with mental health care utilization, impairment, and higher risk for engaging in risky behaviors like substance abuse. As such, they argue that 5 should be the cut-off score for denoting “moderate” mental distress. Among the sample population, about 44% of respondents score 5 or greater on the distress scale, and 8% score 13 or higher.

**Fig 1:**
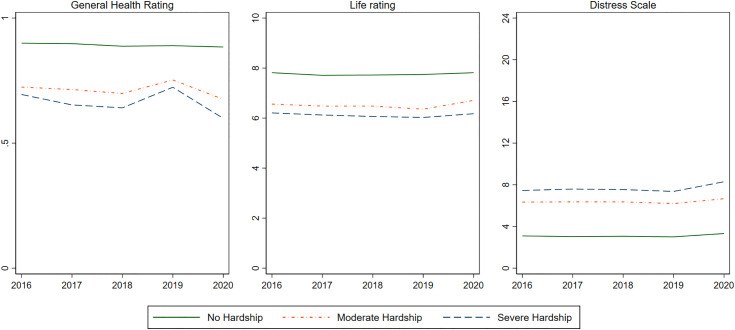
Mean health rating over time by hardship status.

Fig 1 shows the mean rating across health outcomes by whether a respondent experienced no hardship, at least one moderate hardship, or at least one severe hardship. Across the study period, those experiencing severe hardship rate all three health outcomes worse than those who experience moderate hardship, who are also in much worse health than those experiencing no hardship. In percentage terms, those experiencing moderate hardship rate their general health about 19.5% worse and their overall life 16.1% worse. Those experiencing severe hardship rated 22.8% and 20.6% lower on general health and life rating. Numbers were most shocking for distress, where the Kessler score for those facing moderate hardship was 105% higher and 141% higher among those experiencing moderate and severe hardship, respectively. Around 50% of the survey population experienced at least one moderate hardship, and 33% experienced at least one severe hardship over the course of the study period.

### Analytical strategy

The models used in this analysis include logistic regression for the binary general health rating variable, and ordinary least squares (OLS) regression for life rating and distress, to estimate the relationship between material hardship and adverse health outcomes.

The regressions take the following functional form.


$$Health_{ibt}=\beta_{\text{1}}ModerateHardship_{ibt}+\beta_{\text{2}}SevereHardship_{ibt}+X_{ibt}^{\prime }+\gamma_{b}+\delta_{t}+\epsilon_{ibt}$$ (1)

Where *Health* represents three outcome variables: general health rating, life rating, and distress scale. The subscripts i, b, and t represent individual respondents (i), each of the five boroughs in New York City (b), and year of the survey wave from 2016–2020 (t). For each hardship (energy, food, health, housing, and financial), individual regressions are run that include whether a respondent experienced moderate or severe hardship, represented by $\beta_{\text{1}}$ and $\beta_{\text{2}}$. *X’* represents a vector of sociodemographic control variables as detailed below, and $\gamma_{b}$ and $\delta_{t}\;$index the borough and year level fixed effects. These models include fixed effects to account for possible unobserved confounders at the borough level (such as sociodemographic differences and environmental factors), as well as yearly fixed effects to adjust for yearly fluctuations that might influence health outcomes (such as economic conditions). Each model also includes person-level weighting to account for sampling and response biases. Finally, standard errors are clustered at the borough level to account for the likely correlation of within-borough socioeconomic and policy conditions.

The first set of models come from individual regressions of each hardship (e.g., health regressed on energy hardship) that are then combined into coefficient plots to compare their relative effect on the outcome of interest. These models compare the association of health with moderate hardship as well as severe hardship, as defined descriptively in Table 1.

**Table 1:** Descriptive statistics of health outcomes, hardship measures, and controls using person-level weights.

Outcomes	Definition	Obs	Mean	SD	Min	Max
General Health Rating	A binary variable representing a respondent’s ranking of their general health, where 0 = poor or fair, and 1 = good, very good, or excellent	11,397	0.80	0.40	0	1
Life Rating	Respondent’s rating of their life overall on a 10-point scale, ranging from “the worst possible life overall” to “the best possible life overall”	11,332	7.03	1.92	0	10
Distress Scale	A summary measure representing the number of points respondents scored on the psychological distress scale representing how often, in the last 30 days, the respondent felt: nervous, restless or fidgety, hopeless, worthless, that everything was an effort, or so sad that nothing could cheer them up.	11,149	5.09	4.79	0	24
**Regressors**						
Moderate food hardship	Respondent sometimes worried their food wouldn’t last, or sometimes their food did not last in the past 12 months	11,326	0.28	0.45	0	1
Severe food hardship	Respondent often worried their food wouldn’t last, or their food often didn’t last	11,326	0.09	0.29	0	1
Moderate energy hardship	Respondent could not pay their gas, oil, or electricity bill in the past 12 months	11,326	0.13	0.34	0	1
Severe energy hardship	Respondent had their gas or electricity service cut off in the past 12 months	11,326	0.10	0.30	0	1
Moderate housing hardship	Respondent could not pay their full rent/mortgage in the past 12 months	11,326	0.14	0.34	0	1
Severe housing hardship	Respondent moved in with someone else, or into temporary housing, in the past 12 months	11,326	0.06	0.23	0	1
Moderate financial hardship	Respondent sometimes ran out of money in the past 12 months	11,326	0.33	0.47	0	1
Severe financial hardship	Respondent often ran out of money in the past 12 months	11,326	0.13	0.34	0	1
Medical hardship	Respondent or family member could not go to the doctor, dentist, or the hospital because of the cost in the past 12 months	11,326	0.18	0.38	0	1
**Controls**						
Black	Respondent’s race is Black, Non-Hispanic	11,326	0.21	0.41	0	1
Hispanic Other	Respondent’s race is Hispanic Respondent’s race is Asian, Non-Hispanic or Other/Multiracial	11,326 11,326	0.27 0.16	0.44 0.37	0 0	1 1
Female	Respondent identifies as “female”	11,326	0.53	0.50	0	1
Age	Respondent’s age	11,326	46.16	17.41	18	85
Renter	Respondent is a renter	11,326	0.50	0.50	0	1
Household earnings	Total household income	11,326	54,933	65,068	0	276,474
Bedrooms	Number of bedrooms in respondent’s home	10,746	2.31	1.21	0	10
Disability	Respondent has a disability which prevents them from working, or limits the kind/amount of work they can do	11,292	0.21	0.41	0	1
Owe debt	Respondent owes credit card, medical, or “other” debt	11,079	0.52	0.50	0	1
Received assistance	Received housing, SNAP, WIC, Medicaid, retirement, disability, unemployment, or cash assistance in the last 12 months	11,326	0.50	0.50	0	1
Children under 18	Children under the age of 18 live in the respondent’s household	11,326	0.61	1.03	0	8
Household size	Number of household members in the respondent’s home	11,326	2.90	1.63	1	9

Subsequently, two alternative approaches are conducted to analyze the impact of hardship. The first measures the differential association between health and chronic hardship as compared to acute hardship. For each of the five hardships, a respondent is coded as facing acute hardship (food, energy, housing, financial, health) if they experienced that hardship only once during the five-year study period, and chronic if they experienced that hardship more than once. Past literature has found an association between chronic hardship and higher rates of stress and diminished emotional wellbeing [54,55]. Across the sample, roughly 38%, 25%, 20%, 48%, and 18% of respondents chronically experienced food, energy, housing, financial, and medical hardship, respectively.

The second model measures the association between health outcomes and the number of simultaneous hardships that a respondent faces in each survey wave. This variable is constructed as a count variable, ranging from 0 to 5, that adds up the number of hardships a respondent faced in each wave of the survey. To estimate the impact of each additional hardship on health, post regression marginal effect estimates are calculated. Importantly, because the explanatory variable is a count variable, models are estimated using a categorical specification of hardship count, which avoids imposing a linear form to the potential nonlinear impact of each additional hardship.

To summarize the analytic approach, the analysis includes three sets of models. The first measures the association between moderate and severe hardship and health outcomes. The second examines whether chronic hardship is correlated with worse health outcomes compared to acute hardship. The third assesses how additional simultaneous hardships influence health ratings. For each model, the physical health estimate comes from a logistic regression model and the mental health models (life rating and distress) are measured using OLS. To compare estimates, percentage changes are calculated for each estimate alongside the beta coefficients.

## Results

To analyze the impact of experiencing material hardship on health outcomes, food, energy, housing, financial, and medical hardship are measured over the previous 12 months. Apart from a single medical hardship indicator, which asks whether a respondent or their immediate family member could not go to the doctor, dentist or hospital due to cost, the four other hardship indices are included as either “moderate” or “severe.” For food hardship and financial hardship, severity is defined by the frequency of facing food and money shortages. For energy and housing hardship, severity is defined by more extreme consequences, i.e., having your power cut off or having to leave your home. Finally, a lengthy set of control variables is included. These include basic demographic information (race, gender, age, disability status), characteristics of the household (size, renter status, children), and respondent financial status (earnings, debt, assistance).

All variable definitions, along with descriptive statistics, can be found in Table 1. On average, people tend to rate their health relatively well. For general health, where 0 indicates poor or fair health rating and 1 indicates good or better, the mean score is 0.8, meaning most survey respondents rate themselves as in good health. Similarly, the mean life rating was greater than 7. That said, the mean Kessler rating score was right around 5, which Prochaska et al. [53] argue is the cutoff line for moderate distress. In terms of hardship variables, moderate financial hardship and moderate food hardship are by far the most common forms of hardship, with a mean rate of 28% and 33% across survey waves, respectively. Medical hardship is also quite common. A smaller proportion of the survey population experienced severe hardships, with severe housing hardship, indicating at least temporary housing instability, being the rarest hardship. Control variables largely look as expected but also indicate the relatively high level of inequality in New York City. Half of survey respondents rent their home and receive government assistance, and more than half owe some level of debt. The sample is also racially diverse, with 64% identifying their race as non-white.

### Physical health

To analyze physical health, this study uses a measure which asks respondents to rate their general health. In the 2015 wave of the Poverty Tracker survey, a question is included that asks respondents specifically to rate their physical health but is dropped from the survey in subsequent waves. As a test of validity, the same logistic regression model was applied on physical health rating (also measured as a Likert scale and transformed to a binary variable representing poor or good health) among wave 1 respondents. Results, displayed in S1 Fig, are generally consistent with the finding below in terms of direction, magnitude, and significance, suggesting that general health rating serves as a reasonably consistent indicator of physical health.

**Fig 2:**
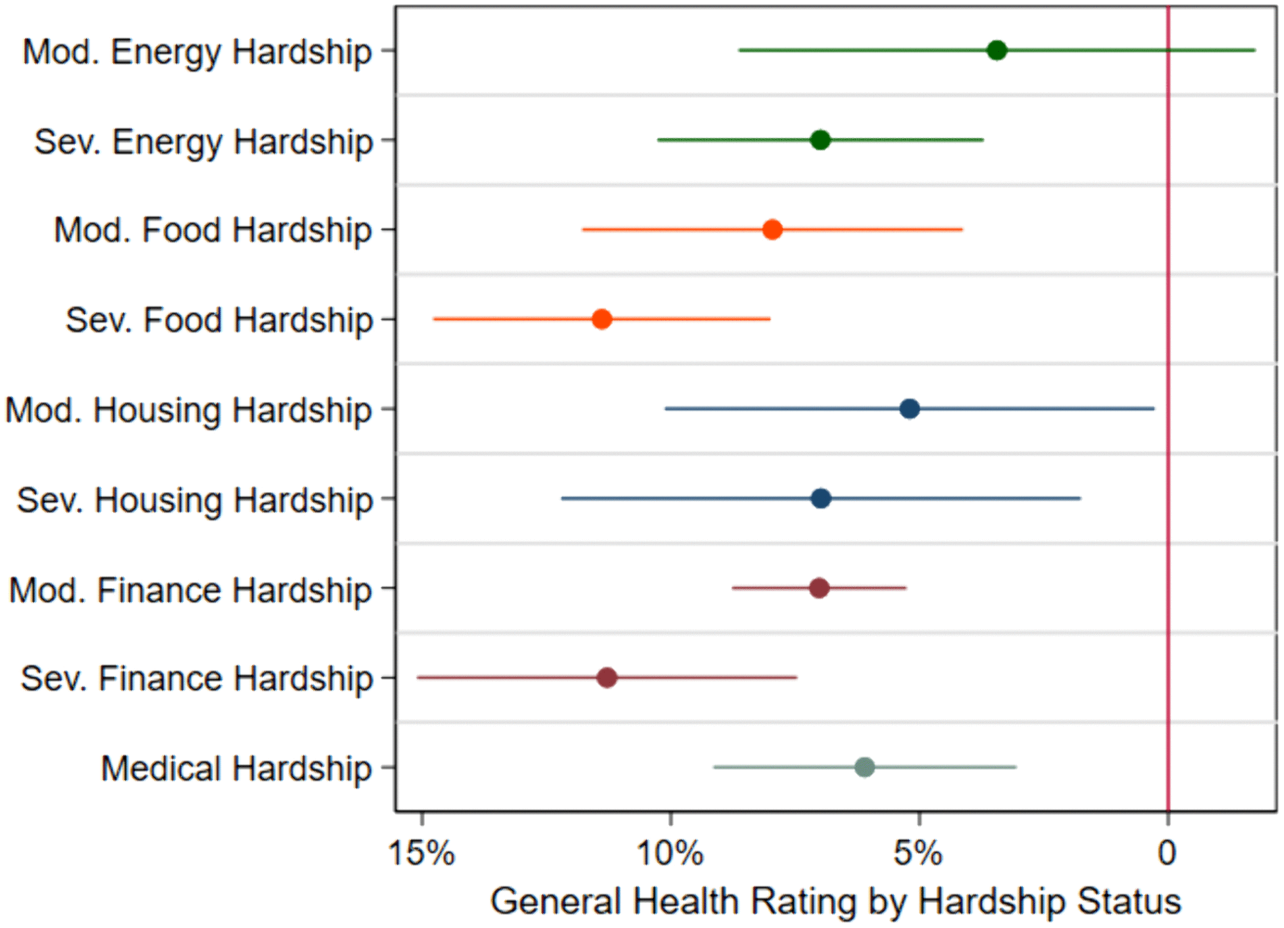
Average marginal effect plot of moderate and severe hardship on general health rating. Figure displays logistic regression coefficients, with standard errors clustered at the borough level and 95% confidence intervals. N = 10,493.

Fig 2 displays results using the general health measure. Each moderate and severe hardship, except for moderate energy hardship, is significantly associated with a lower probability of a respondent reporting good health. To aid interpretation of effect size, average marginal effects (AMEs) are estimated following each logistic regression models. These AMEs represent the mean percentage point change in the probability that a respondent reports good health. The largest effect is among those experiencing severe food hardship, who reported a 11.4 percentage point decrease in the probability of reporting good health (β = −0.99, p < 0.001).

In each instance, severe hardships are associated with a greater reduction in the probability of reporting good health than moderate hardships, although each estimate falls within overlapping 95% confidence intervals. The variation in the size of the gap between these effects is notable. Controlling for socio economic factors, the association between severe housing hardship (moving at least temporarily) and general health is a 1.8 percentage point greater reduction in the probability of reporting good health compared to moderate housing hardship (not being able to full pay rent or mortgage), with respective coefficients of β = −0.59 (p = 0.008) and β = −0.44 (p = 0.036). For financial hardship, the difference is more pronounced, where severe financial hardship (often running out of money) is associated with a 4.2 percent larger reduction in the probability of reporting good health than moderate financial hardship (sometimes running out of money), with coefficients of β = −0.98 (p < 0.001) and β = −0.61 (p < 0.001), respectively. Severe food hardship has the strongest association, consistent with S1 Fig, indicating that food insecurity is most strongly associated with diminished physical health, especially when one regularly runs out of food.

### Mental health

The analysis next examines the mental health impacts of material hardship, measuring respondent life rating and level of distress. As in the results above, coefficients from these models are converted to percentages changes by dividing regression coefficients by the maximum of the variable scale (10 for life rating, and 24 for the Kessler Scale of distress). For example, a coefficient of −1.0 in life rating corresponds to a 10% decrease.

**Fig 3:**
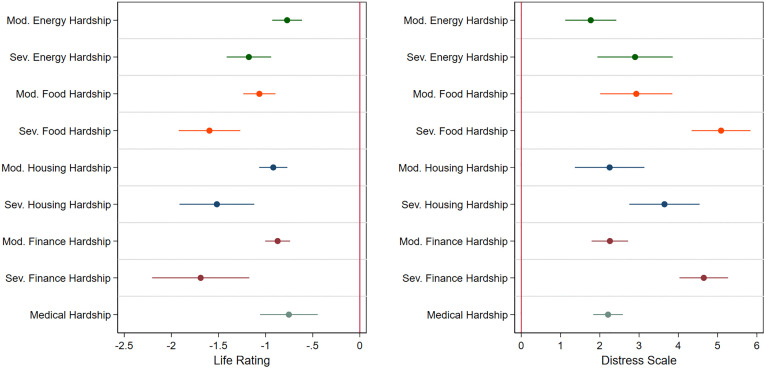
Coefficient plot of moderate and severe hardship on life rating and distress. Figure displays OLS regression coefficients, with standard errors clustered at the borough level and 95% confidence intervals. N = 10,547.

Results, shown for both models in Fig 3, reveal that all forms of moderate and severe hardship have a statistically significant impact on mental health. The magnitude of the associations on a respondent’s life rating range from a 7.5% decrease (medical hardship; β = −0.75, p < 0.001) to a 16.9% decrease (severe financial hardship; β = −1.69, p < 0.001). The corresponding associations for respondent level of distress range from a 7.4% increase (moderate energy hardship; β = 1.77, p = 0.001) to 21.2% increase (severe food hardship; β = 5.09 p < 0.001).

Of note, severe food hardship is associated with a greater than 5-point increase, on average, on the Kessler Scale of distress, which itself is the threshold for moderate mental distress established by Prochaska et al. [53]. This suggests that, even absent the myriad other life factors that may cause someone distress, facing severe food hardship is likely to lead to mental distress levels that are associated with significant detrimental health outcomes.

As in the case of physical health impacts, moderate energy hardship has the weakest effect on mental health and severe food hardship is among the strongest contributors to a worse life rating and higher level of distress. Unlike the physical health model, the effect sizes and confidence intervals of both mental health models generally show statistical distinctions between severity of hardship outside of 95% CI overlaps, where severe hardships are much more strongly associated with a decline in mental health rating. For example, a respondent reporting that they often run out of money (severe financial hardship) had more than twice the impact on distress levels than that of a respondent saying that they only sometime run out of money (moderate financial hardship). When compared to Fig 2, these results may suggest that material hardships are more strongly associated with mental health outcomes, and further that these associations more strongly scale with severity of hardship.

### Acute and chronic hardship

To further interrogate the effect of hardship on health, this section compares the relationship between acute versus chronic hardship, as defined above, and health outcomes. Results appear in Table 2. As above, for the logistic regression model, marginal effect estimates are displayed for ease of interpretation.

**Table 2:** Logistic and ordinary least squares regressions predicting health outcomes by acute and chronic material hardship.

	General Health Rating	Life Rating	Distress Scale
Acute Food Hardship	−0.042^***^	−0.308	1.627^**^
	(0.011)	(0.136)	(0.329)
Chronic Food Hardship	−0.098^***^	−1.273^***^	3.787^***^
	(0.018)	(0.033)	(0.369)
Acute Energy Hardship	−0.043^***^	−0.544^*^	0.916
	(0.015)	(0.150)	(0.371)
Chronic Energy Hardship	−0.064^***^	−1.119^***^	2.625^***^
	(0.017)	(0.050)	(0.301)
Acute Housing Hardship	−0.016	−0.353	1.538^**^
	(0.012)	(0.145)	(0.196)
Chronic Housing Hardship	−0.075^*^	−1.233^***^	3.133^***^
	(0.036)	(0.057)	(0.318)
Acute Financial Hardship	−0.040	−0.389	1.135
	(0.027)	(0.177)	(0.507)
Chronic Financial Hardship	−0.093^***^	−1.088^***^	3.075^***^
	(0.013)	(0.065)	(0.100)
Acute Medical Hardship	−0.047^***^	−0.491^*^	1.409^**^
	(0.009)	(0.113)	(0.224)
Chronic Medical hardship	−0.080^***^	−0.907^***^	2.772^***^
	(0.026)	(0.051)	(0.272)
Controls? Fixed Effects?	Yes Yes	Yes Yes	Yes Yes
Observations	10,493	10,448	10,358

Robust standard errors in parentheses *** p < 0.001, ** p < 0.01, *p < 0.05.

When compared to previous findings, Table 2 makes clear that the effects on health are largely driven by the frequency of hardship that a household faces. For each of the five hardships, the effect of chronic hardship is substantially greater than that of acute hardship, and each finding is statistically significant. Consistent with findings above, chronic food hardship has the strongest impact on all three outcomes. In percentage terms, chronic food hardship was associated with a 9.8 percentage point decrease in the probability of reporting good health (β = −0.816, p < 0.001), a 12.7% decrease in life rating (β = −1.27, p < 0.001), and a 15.8% increase in reported distress (β = 3.79, p < 0.001).

The difference between acute and chronic hardship is particularly notable for a respondent’s physical health. The coefficients on acute housing and financial hardship in the general health rating model are not statistically significant, while the marginal effects for the chronic measures are more than twice as large, and statistically significant. Overall, these findings suggest that chronic hardship is considerably more strongly associated with diminished physical health outcomes than acute hardship, perhaps because adverse physical health outcomes (e.g., hypertension, obesity, untreated medical conditions) tend to take their toll when accumulated over time through chronic deprivation.

### Number of hardships

**Fig 4:**
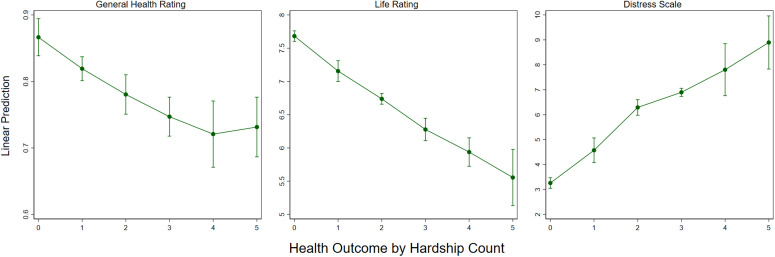
Marginal effects from logistic and ordinary least squares regression of count of material hardships on health outcomes. Figure displays marginal effects from an OLS regression model, with standard errors clustered at the borough level and 95% confidence intervals. N = 10,547.

The final set of models estimate health outcomes by the number of hardships that a respondent faces. Across the sample, 42% experienced no hardship at all, while 14.6%, 14.7%, 12.2%, 10.7%, and 5.2% experienced 1, 2, 3, 4, and 5 hardships, respectively. These percentages stay roughly consistent over the course of the 5-year study period. It is notable that facing 5 hardships is not a particularly rare occurrence, encompassing 589 out of 11,402 total survey respondents across waves, and never making up less than 4.8% of the survey population in any year. Results are displayed in Fig 4.

On average, each additional hardship is associated with a 3.6 percentage point lower likelihood of reporting good health, a 4.2 percent decrease in life satisfaction, and a 4.7 percent increase in psychological distress, measured relative to population means. However, Fig 4 also shows that the relationship is not strictly linear. Life rating tends to decline steadily with each additional hardship, whereas that associations with physical health and distress appear to level off after the third hardship.

### Robustness checks

Although the above models include disability status as a control – specifically a question that asks respondents if they have a disability that limit them from working – there is potential for reverse causality in the findings, namely that poor health may impact the likelihood that an individual faces material hardship. To address this concern, models are run in S1 Table that include a 1-year lag of each form of hardship. Results show that each lagged variable is strongly significant. This strong association between experiencing hardship in the previous year and current health rating provides additional evidence that hardship is in fact impacting health outcomes and not the reverse.

Further, there may be concern that, across the three health models, there are common unobserved factors that influence results in the same direction (like chronic health conditions), leading to biased estimates. To account for this, Seemingly Unrelated Regression (SUR) models are run to model potential interdependence and resulting correlation in error terms across all three outcomes. Results, displayed in S2 Table, remain robust and are similar to the estimates in Fig 2 above, suggesting that results are not substantively driven by unobserved factors jointly affecting health outcomes.

## Discussion

This study provides estimates of the association between material hardship and health. Examining longitudinal data on New York City residents, the analysis finds a sizable and persistent correlation between each form of material hardship and diminished physical and mental health outcomes, even after controlling for a lengthy set of sociodemographic covariates. For both physical health and mental health, severe hardships have a greater negative impact than moderate hardships. Severe food and financial hardship are most strongly correlated with a diminished health rating. On average, those facing severe food hardship rated their general health 9.8 percentage points worse, their overall life rating 12.7 percent lower, and scored 15.8 percent higher on the Kessler Scale of psychological distress.

Next, the frequency with which respondents face hardship is examined. For each hardship, health outcomes for those experiencing chronic hardship are substantially worse than for those who experience hardship acutely. Across all three health measures, for example, the association with chronic financial hardship is more than twice as great as with acute financial hardship. The statistical association between physical health and hardship is particularly sensitive to frequency, suggesting that effects of experiencing hardship may accumulate over time to increase physical risk factors.

Finally, health outcomes are measured based on the number of hardships a respondent faces simultaneously. Each additional hardship decreases a respondent’s general health rating by roughly 3.6 percentage points, lowers their life rating by 4.2 percent, and increases their distress by almost 5 percent, on average. An individual facing 5 material hardships simultaneously, comprising more than 5% of the survey population, rates their general health rating, life rating, and distress about 13 percentage points, 21 percent, and 24 percent worse, respectively, than those experiencing no hardship. With respect to the Kessler Scale, results suggest that the increase from experiencing 1 hardship to experiencing 2 hardships simultaneously is, on average, associated with putting an individual into the classification of “moderate mental distress,” which correlates with significant adverse health outcomes that are predictive of the need for medical treatment [49,53].

This analysis has several limitations. First, the study includes an imperfect measure of physical health. Although the measure is considered valid in the literature (see [56]) and it is consistent with the survey item asking directly about physical health rating in wave 1 of the survey, asking a respondent to rate their general health may be open to interpretation. Relatedly, the survey does not include actual health outcomes, such as asthma or hypertension, that could give a more nuanced picture of the impact of specific hardships on health. Third, the difference in scale between the outcomes does not necessarily allow for precise effect-size comparisons, which is limited by the possibility that a survey respondent may rate a Likert scale item differently than they rate a 0–10 ranking, as suggested by research in psychology (see, e.g., [57]). The final limitation is one of external validity. New York City presents a relatively unique case study compared to many other cities in the United States, with a notably more diverse population and a higher cost of living than much of the country. While the overall finding that experiencing material hardship substantially impacts health is consistent in the literature, the relative effect size of each type of hardship may differ in other research settings. For example, the finding that food insecurity is most strongly associated with worsened health outcomes may be partially driven by the fact that the average meal cost in New York County is nearly twice as high as the national average, according to a 2022 report from Feeding America [58].

The findings of this study have important implications for scholars and policymakers. Results suggest that the experience of material hardship – which regularly impact millions of American households – is significantly associated with diminished health. This highlights the important role of funding, expanding, and better targeting social welfare programs. Such an approach might include expansion of federal programs like SNAP and housing programs, where expanded access has been associated with a reduction in hardship [59–61], grant programs for local governments who have shown effectiveness with programs like Housing First [62], and/or incentives for nonprofit organizations that alleviate hardship through measures like meal services and utility bill assistance [63,64].

Because material hardship is often chronic, policymakers should focus on solutions that are preventative rather than just remedial. Some examples include weatherizing homes, expanding access to solar energy [65], low-cost medical screenings, low-income tax credits, basic income programs [66], and reduced or free school lunch programs [67]. Such approaches would reduce the incidence of material hardship but also likely mitigate the secondary effect of cost and strain on the medical system.

Future scholarship should expand on the results found in this study by conducting similar longitudinal analysis to contexts outside of New York City to compare the effects of material hardship on health in different socioeconomic contexts. Impacts might differ based on geographic heterogeneity (e.g., rural areas), economic conditions (e.g., prices), and policy (e.g., social services). Additionally, future research could focus on the cumulative effect of hardship over a longer span of time, providing insights into longer-term health trajectories that result from persistent hardship. Finally, integrating more precise physical health data could improve the validity of outcome measures and allow for a more nuanced picture of how type, severity, and frequency of material hardships comparatively influence health outcomes.

## Supporting information

S1 Fig Coefficients from a logistic regression of moderate and severe hardship on physical health rating. (TIFF)

S1 Table Logistic and ordinary least squares regression predicting health outcomes, including a lag of each type of material hardship. (DOCX)

S2 Table Seemingly unrelated regression predicting health outcomes. (DOCX)
