# Supplementary material for: The impact of material hardship severity and frequency on health outcomes: Evidence from New York City
Source: PLoS One. 2025 Oct 30;20(10):e0335790. doi: 10.1371/journal.pone.0335790 (PMC12574881; doi:10.1371/journal.pone.0335790)
Supplement: S1 Table — (DOCX) [file pone.0335790.s002.docx]

**S1 Table. Logistic and Ordinary Least Squares Regression Predicting Health Outcomes, Including a Lag of Each Type of Material Hardship**

|  | General Health Rating | Life Rating | Distress scale |
| --- | --- | --- | --- |
|  |  |  |  |
| Energy hardship (1-year lag) | -0.280*** | -0.398*** | 0.600*** |
|  | (0.097) | (0.058) | (0.153) |
| Energy hardship | -0.286 | -0.736*** | 1.927*** |
|  | (0.217) | (0.069) | (0.247) |
| Food hardship (1-year lag) | -0.423*** | -0.465*** | 1.631*** |
|  | (0.027) | (0.072) | (0.303) |
| Food hardship | -0.519*** | -0.913*** | 2.469*** |
|  | (0.129) | (0.044) | (0.281) |
| Housing hardship (1-year lag) | -0.217** | -0.460*** | 1.102*** |
|  | (0.084) | (0.063) | (0.239) |
| Housing hardship | -0.387* | -0.872*** | 2.127*** |
|  | (0.208) | (0.028) | (0.306) |
| Financial hardship (1-year lag) | -0.476*** | -0.402*** | 1.357*** |
|  | (0.050) | (0.063) | (0.111) |
| Financial hardship | -0.434*** | -0.872*** | 2.082*** |
|  | (0.083) | (0.028) | (0.082) |
| Medical hardship (1-year lag) | -0.437*** | -0.334 | 1.476*** |
|  | (0.111) | (0.163) | (0.225) |
| Medical hardship | -0.339** | -0.606*** | 1.580*** |
|  | (0.152) | (0.127) | (0.192) |
| Observations | 10,486 | 10,441 | 10,352 |

Robust standard errors in parentheses *** p<0.01, ** p<0.05, *p<0.1
