# Supplementary material for: The impact of material hardship severity and frequency on health outcomes: Evidence from New York City
Source: PLoS One. 2025 Oct 30;20(10):e0335790. doi: 10.1371/journal.pone.0335790 (PMC12574881; doi:10.1371/journal.pone.0335790)
Supplement: S2 Table — (DOCX) [file pone.0335790.s003.docx]

**S2 Table. Seemingly Unrelated Regression Predicting Health Outcomes**

|  | General Health Rating | Life Rating | Distress scale |
| --- | --- | --- | --- |
|  |  |  |  |
| Moderate energy hardship | -0.044*** | -0.778*** | 1.728*** |
|  | (0.010) | (0.053) | (0.127) |
| Severe energy hardship | -0.100*** | -1.202*** | 3.056*** |
|  | (0.012) | (0.062) | (0.149) |
| Moderate food hardship | -0.079*** | -1.060*** | 2.586*** |
|  | (0.009) | (0.044) | (0.103) |
| Severe food hardship | -0.203*** | -1.720*** | 5.259*** |
|  | (0.013) | (0.064) | (0.151) |
| Moderate housing hardship | -0.069*** | -0.851*** | 1.958*** |
|  | (0.010) | (0.052) | (0.126) |
| Severe housing hardship | -0.103*** | -1.490*** | 3.541*** |
|  | (0.017) | (0.084) | (0.202) |
| Moderate financial hardship | -0.069*** | -0.816*** | 2.052*** |
|  | (0.009) | (0.043) | (0.101) |
| Severe financial hardship | -0.174*** | -1.679*** | 4.771*** |
|  | (0.012) | (0.058) | (0.137) |
| Medical hardship | -0.072*** | -0.769*** | 2.086*** |
|  | (0.009) | (0.048) | (0.115) |
| Observations | 10,326 | 10,326 | 10,326 |

Robust standard errors in parentheses *** p<0.01, ** p<0.05, *p<0.1
